# Influence of letter shape on readers’ emotional experience, reading fluency, and text comprehension and memorisation

**DOI:** 10.3389/fpsyg.2023.1107839

**Published:** 2023-02-15

**Authors:** Tanja Medved, Anja Podlesek, Klementina Možina

**Affiliations:** ^1^Faculty of Natural Sciences and Engineering, University of Ljubljana, Ljubljana, Slovenia; ^2^Faculty of Arts, University of Ljubljana, Ljubljana, Slovenia

**Keywords:** typeface shape, pleasantness, reading fluency, text comprehension, text memorisation, age differences

## Abstract

**Introduction:**

The amount of educational material delivered to pupils and students through digital screens is increasing. This method of delivering educational materials has become even more prevalent during the COVID-19 pandemic. To be as effective as possible, educational material must be properly designed not only in terms of content, but also in terms of form, e.g., the typeface. The present study investigated the effect of letter shape on readers’ feelings of pleasantness during reading, reading fluency, and text comprehension and memorisation.

**Methods:**

To find out whether age influences the effects of typeface shape on reading measures, we divided the participants into a group of less experienced readers (children) and more experienced readers (adults). Both groups read texts in eight different typefaces: four of them were round or in rounded shape, and four were angular or in pointed shape. With an eye-tracker, the reading speed and the number of regressive saccades were recorded as measures of reading fluency and changes in pupil size as an indicator of emotional response. After reading each text, the participants rated the pleasantness of the typeface, and their comprehension and memorisation of texts were checked by asking two questions about the text content.

**Results:**

We found that compared to angular letters or letters in pointed shape, round letters or letters in round shape created more pleasant feelings for readers and lead to a faster reading speed. Children, as expected, read more slowly due to less reading experiences, but, interestingly, had a similar number of regressive saccades and did not comprehend or remember the text worse than university students.

**Discussion:**

We concluded that softer typefaces of rounder shapes should be used in educational materials, as they make the reading process easier and thus support the learning process better for both younger and adult readers. The results of our study also showed that a comparison of findings of different studies may depend on the differences among the used letter shapes.

## 1. Introduction

The shape of letters and the general typographic design of a text affect the legibility of the text (Beier at al., 2017), the transparency of the presentation of information (Brath and Banissi, 2016) and, consequently, the fluency of reading (Gasser et al., 2005; Beier and Larson, 2013; Cacali, 2016; Bessemans, 2016a,b). The present study examined the effect of letter shape on readers’ feelings of pleasantness during reading, pupil size and eye movements during reading, and text comprehension and memorisation.

### 1.1. Reading fluency

The concept of reading fluency combines accuracy and speed of reading with the ability to comprehend the content being read. Some definitions of reading fluency focus more on letter recognition and reading speed (Meyer and Felton, 1999), while others include content comprehension (Pikulski and Chard, 2005).

Many factors affect reading fluency. Reading fluency is affected by the shape or legibility of the typeface (Ali et al., 2013), type size (Mueller et al., 2014; Su et al., 2018), and the overall typographic design of the text (Koch, 2012). When reading on a screen, reading fluency is also affected by the screen resolution, as with a higher resolution, letters and their features can be displayed better (Bessemans, 2016a; Bigelow, 2019).

### 1.2. Text comprehension and memorisation

Several studies showed that the shape of letters and the text can influence the comprehension of the read content (Choi et al., 2018) and the actual memorisation of the read content (Lewis and Walker, 1989; Gasser et al., 2005). Poorer fluency results in poorer information processing and, consequently, poorer comprehension and memorisation of the text (Novemsky et al., 2007; Oppenheimer and Frank, 2008; Meyer et al., 2015; Bjork and Yue, 2016; Pieger et al., 2016; Rummer et al., 2016; Sanchez and Nayor, 2018; Dressler, 2019; Wu et al., 2019).

Studies examining how using a perceptually difficult-to-process typeface with an increased desirable difficulty designed specifically to reduce legibility, such as Sans Forgetica, found either no processing or memory benefit of such typefaces or even yielded a memory cost (Geller et al., 2020; Taylor et al., 2020; Wetzler et al., 2021; Cushing and Bodner, 2022; Maxwell et al., 2022). However, there is also a whole series of studies which showed that poorer fluency of the text or desired difficulty in the fluency of the text resulted in better processing of the text and consequently in better memorisation of the read content (Diemand-Yauman et al., 2011; Macdonald and Lavic, 2011; Bjork et al., 2013; Halin, 2016; Pieger et al., 2016).

Numerous studies demonstrated that reading fluency affects the learning process, more specifically short-term and long-term memory (Weissgerber and Reinhard, 2017), as well as metacognition (Yue et al., 2013; Ilic and Akbulut, 2019). Based on the shape of letters and the text, readers can predict how long it will take them to read the text and remember the content of the text (Beier and Larson, 2013; Price et al., 2016). Higher reading fluency should promote a positive attitude towards the text, consequently the feeling of better memorability of the text, and it should allow for better memorisation and comprehension of the text (Song and Schwarz, 2008; Mueller et al., 2013; Labro and Pocheptsova, 2016; Pieger et al., 2016; Mead and Hardesty, 2018). In contrast, poorer fluency should promote poorer attitudes toward the text and readers should assume that they will spend more time reading and memorising the text.

### 1.3. The role of emotions in the reading process

Emotions play a specific role in reading. The typographic design or the shape of the typeface has a great impact on the reader’s mood, more specifically on their emotional response or feeling of pleasantness that the reader experiences when reading certain letterforms (Larson and Picard, 2005; Larson et al., 2006; Koch, 2012; Petit et al., 2015). The shape of letters and the text can suggest the nature and content of the text to the reader (Lewis and Walker, 1989; Ehsen and Lupton, 1998; Celhay et al., 2015; Bigelow, 2019; Davis, 2019; Raden and Qeis, 2019).

Several studies have shown that the perception of shapes, tastes and sounds evokes various feelings in humans, including the feeling of pleasantness (Childers and Jass, 2002; Brumberger, 2003; Mackiewicz, 2005; Shaikh et al., 2006; Bar and Neta, 2007; Tsonos and Kouroupetroglou, 2011; Amare and Manning, 2012; Crisinel et al., 2012; Ngo et al., 2013; Velasco et al., 2014, 2015a,b, 2016, 2018a; Salgado-Montejo et al., 2015; Jordan, 2017; Davis, 2019; Haenschen and Tamul, 2019). Round and rounded shapes, as well as symmetric shapes evoke more pleasant feelings than angular or pointed and asymmetric shapes (Bar and Neta, 2007; Ngo et al., 2013; Turoman et al., 2018; Velasco et al., 2018b).

We have not found a study that would examine how these features of human perception can be effectively used in the typographic design of educational materials, but based on the previous studies we can assume that round typefaces would evoke more pleasant feelings than angular ones.

### 1.4. The influence of letter shape on the reading process

The core of typographic design are typefaces, which can be grouped based on the shape of the main strokes, and the transitions between the strokes and the stroke ends (terminals, serifs). One group of typefaces contains round/rounded typefaces and the other group contains angular/pointed typefaces. A typical example of typefaces that could be classified in the round/rounded group based on their design features are typefaces that belong to the group of Venetian, Garalde and Transitional typefaces (McLean, 1997; Možina, 2003). Typefaces that could be classified in the angular/pointed group based on their design characteristics are typefaces that belong to the Didone, Slab-Serif and Sans Serif group (McLean, 1997; Možina, 2003).

Previous studies found that rounded, organic shapes of strokes and softer transitions between the strokes and stroke ends are perceived as more pleasing whereas the letters with more geometric stroke shapes, sharp transitions between strokes and finial stroke are found to be less pleasant (Spence and Deroy, 2012; Hyndman, 2016). The feeling of pleasure we experience when reading different typefaces influences motivation and concentration (Mano, 1997; Koch, 2012), memorisation and comprehension of a text (Mano, 1997). However, research addressing how the reader’s emotional response to the shape of letters affects reading fluency is scarce.

### 1.5. The effect of age on reading

It has been shown that perception in reading also depends on the age of the reader. Children and adult readers differ in the level of development of cognitive and physiological abilities until the age of four, after which the ability to recognise letters should be the same in children and adults (Woods et al., 2005). However, studies reported that children from 4 to 11 years old react to different stimuli, e.g., colour, shape, taste, smell, differently from adult students (Gollely and Guichard, 2011). It has been discovered that reactions to the same stimuli are different also in younger adults (under 35 years old) and older adults (over 60 years old) (Piqueras-Fiszman et al., 2011). In younger readers (aged 7 to 9 years), typefaces with serifs and a difference in stroke width were found to lead to more fluent reading, whereas sans serif typefaces that have no or minor difference in stroke width result in fewer reading errors (Wilkins et al., 2009). It is also claimed that a larger type size allows faster decoding of information and better memory, but only in children (age 9 to 12 years old), not in adult students (Abukaber and Lu, 2012). Children (from 7 to 12 years old) read letters that are heterogeneous in shape more easily (Wilkins et al., 2009; Abukaber and Lu, 2012); especially the heterogeneity in the shape of letters seems to greatly aid visually impaired children (age 5 to 10 years old) in reading (Bessemans, 2016b). In the study conducted by Katzir et al. (2013), the increased desirable difficulty of the typeface affected reading fluency, demonstrating positive effects in older children (11 years old), but negative effects in younger children (8 years old).

### 1.6. The aim of our study

Our study had two aims. The first aim was to determine how the shape of the typeface (round/rounded vs. angular/pointed) affects reading fluency, subjective reading experience, and reading performance. The second aim of our study was to investigate whether the effect of typeface shape is the same for younger, less experienced readers and for adult, more experienced readers.

We used an eye-movement tracking device as it provides objective measures of reading fluency (Piqueras-Fiszman et al., 2013; Franken et al., 2015). We monitored the reading speed and regressive saccades as measures of reading fluency. We also used this device to observe changes in pupil size, which should be indicative of the reader’s emotional response (Hess and Polt, 1960; Margareth et al., 2008; Wang et al., 2018). Objective measures of emotional response to different shapes of typefaces were complemented with subjective ratings of feelings of pleasantness. Text comprehension and memorisation of what was read were also observed as indicators of reading performance.

## 2. Methods and materials

The studies involving human participants were reviewed and approved by the Ethics Commission of the Faculty of Arts, University of Ljubljana. An informed consent document to participate in this study was provided by the participants or their legal guardian/next of kin. All studies were performed in accordance with the Declaration of Helsinki.

### 2.1. Apparatus

To track eye movements, we used a Tobii X120 eye-tracking device and Tobii Studio 3.4.8 software (Tobii AB, Sweden). The device tracks eye movements by tracking the reflection of the image from the cornea. The corneal reflection is generated by infrared emitters on the front of the device that create IR light patterns that are then reflected off the cornea. The device contains a camera that is sensitive to IR light and monitors each movement and fixation of the eye based on the reflection of IR light from the cornea (Tobii Pro, 2017).

Before the measurements, each participant had 5 min to adapt to the lighting conditions in the test room and to perform a nine-point screen-based calibration of the device. We used an LCD screen with a resolution of 2,400 × 1900 pixels (pixel size 0.27 mm) and a refresh rate of 60 Hz.

### 2.2. Preliminary studies

Prior to the main study in which we investigated how the shape of different typefaces affects the pleasantness ratings and the reading speed, memorisation and understanding of a text, we conducted two preliminary studies. The purpose of the first preliminary study was to select eight texts comparable in cognitive load and the purpose of the second preliminary study was to select eight typefaces.

The measurements were done in a quiet room with walls painted with grey matte paint in accordance with the ISO 3664 standard (ISO 3664, 2009). The letters of the texts that the participants read on the screen were dark on a light background (text colour: #000, background colour: #eee) according to the ISO 12646 standard (ISO 12646, 2015). The participants were located at a distance of 60 cm +/− 1 cm from the screen, in line with the recommendations of the ISO 9241-303 standard (ISO 9241-303, 2011). Their movements were not restricted, but they were asked to remain at a fixed position.

#### 2.2.1. First preliminary study

With the first preliminary study, we selected texts for the main study. Thirty-one students and employees of Faculty of Natural Sciences and Engineering at the University of Ljubljana participated in the study. Their mean age was 44.2 years (*SD* = 7.4), 22 were female and 9 were male. They were not paid for their participation in the study. They reported normal or corrected-to-normal vision.

We prepared 45 different texts in Slovenian (participants’ native language) with contents of similar complexity. The texts were (i) sample texts published as a part of guidelines developed for teachers on how to evaluate the reading efficacy in children (Pečjak and Kramarič, 2018) and (ii) excerpts from a children’s illustrated encyclopaedia about animals (Burnie, 2010). The selected texts had a meaningful beginning and end. They had a length of 457 to 510 characters without spaces (*SD* = 13.55). They appeared on the screen in 10 or 11 lines (*SD* = 0.43) in the Verdana typeface, type size 16 pixels. The text was displayed as an HTML document using the CSS programming language. In this way, we were able to ensure that the text was always displayed in exactly the same type size and position on the screen (i.e., in the centre of the screen).

After calibration, the 45 texts were presented in the same order to all the participants. Consecutive texts were invoked by a mouse click. For each text, we measured the reading speed and the number of fixations.

From the 45 texts, we selected 8 texts for the main study that showed highest reading speeds. They contained 471–510 characters (*M* = 492, *SD* = 18). The average reading speed of the selected 8 texts across the participants varied between 50.39 ms and 56.30 ms per character (*M* = 52.45 ms, *SD* = 2.20 ms). We also examined the number of fixations for each text as another indicator of reading fluency. The lower the number of fixations, the more fluently the participants read the text. The average number of fixations per character varied between 0.35 and 0.44 (*M* = 0.38, *SD* = 0.03). The texts seemed comparable in content complexity and suitable for fluent reading of the general population, including children, and contained no distracting factors such as overly long and demanding words and unclear content. The comparable content difficulty, reading speed and relative number of fixations across the eight selected texts lead us to believe that the texts will result in a similar cognitive load when presented in the main study. The texts and their English translations can be found in the Supplementary Materials.

#### 2.2.2. Second preliminary study

With the second preliminary study, we collected different typefaces for the main study. Fifty-five participants were included, 34 of whom were university students from the same institution as in the first preliminary study. They were between 19 and 26 years old, with the average age of 20.7 years (*SD* = 1.3). Twenty-three were female and 11 were male. The remaining 21 participants were second-triad primary school pupils aged 10 to 12 years, with the average age of 10.7 years (*SD* = 0.6). Ten of them were female and 11 were male. All participants had normal or corrected-to-normal vision and were not paid for their participation in the study.

We checked pleasantness of 15 different typefaces (i.e., Adobe Caslon Pro, American Typewriter, Anka, Arial Nova, Birch STD, Chaparral Pro, Comic Sans MS, Didot, Erlenmeyergraphy, FG April Trial, Matilda, Nogomet, Sans Forgetica, Times New Roman, Verdana). The participants read 15 pangrams on the screen. A pangram is a sentence or a portion of a text that uses all the letters of the alphabet and is typically difficult to read since the content of the sentences formed is unusual or senseless. Each pangram was displayed in a different typeface. The average length of a pangram was 36.9 characters without spaces (*SD* = 3.7). The pangrams were displayed in the centre of the screen. Consecutive texts were invoked by a mouse click.

**Figure 1:**
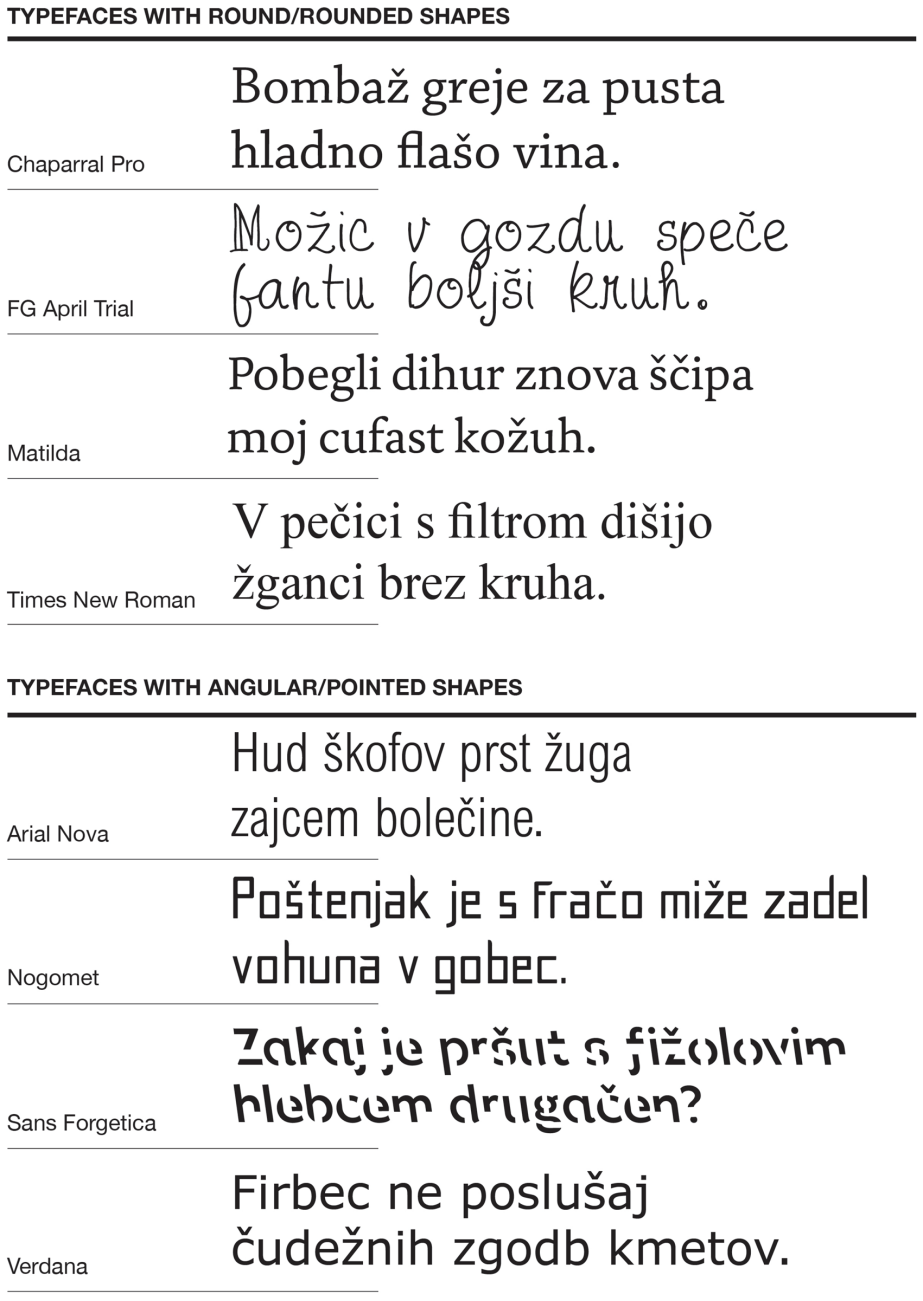
Eight typefaces selected for the main study.

Using a 5-level hedonic scale, participants rated how pleasant they found each typeface (1 – very unpleasant, 2 – unpleasant, 3 – neutral, 4 – pleasant, 5 – very pleasant). Based on the results, we selected eight final typefaces: four rated as most pleasant and four as least pleasant. The four most pleasing typefaces were Chaparral Pro, FG April Trial, Matilda and Times New Roman. These typefaces all had round/rounded shapes: the transitions between the strokes and the stroke ends (terminals, serifs) were soft, just like the transitions between the thick and thin strokes. Also, the shape of the bowls and counters was round and more convex, which is why we considered them as members of the group of round/rounded typefaces. The four least pleasing typefaces had the characteristics of angular/pointed shapes, i.e., Arial Nova, Nogomet, Sans Forgetica, Verdana. These typefaces were all sans serif typefaces, all of them had angular or pointed shaped stroke ends (terminals) and none or minor difference between the thick and thin strokes. The shape of the bowls and counters, especially on the left end right side of the bowl, was less convex and more straight, which is why we considered them as members of the group of angular/pointed typefaces. Figure 1 shows examples of all eight typefaces that we selected for use in the main study.

### 2.3. Main study

#### 2.3.1. Participants

Twenty university students (adult readers; 7 male, 13 female) aged between 18 and 26 (*M* = 20.0 years, *SD* = 1.8 years) and 15 children (pupils of grades 4 to 6 of primary school; 9 male, 6 female) aged 10 through 11 (*M* = 10.7 years, *SD* = 0.5 years) participated in the main study. All participants had normal or corrected-to-normal vision and were not paid for their participation.

#### 2.3.2. Stimuli

For the main study, we used eight selected texts from the first preliminary study and eight selected typefaces from the second preliminary study. Each text was set in one of the typefaces. The texts in different typefaces are shown in the Supplementary Materials.

The size of the typeface was adjusted to achieve the most uniform x-height across typefaces possible, which varied between 0.17 and 0.20 degrees of visual angle; the average x-height was 0.19 degrees of visual angle (*SD* = 0.016). Due to the different shape of the letters, the number of lines of different texts varied between 10 and 11, and the average number of lines was 10.13 (*SD* = 0.35). In all cases, the leading (i.e., line spacing) was 140% of the type size.

#### 2.3.3. Procedure

The main study was conducted under the same standardized conditions as in the first and second preliminary studies. The exception was the lighting in the room, which was now a bright light room, with artificial lighting.

To control for the effect of fatigue, each participant read the texts in a different order (the so-called Latin square). We measured the reading speed, number of saccades, length of fixations, and the size of the pupils for each text in all participants during the whole reading time.

After reading the text on the screen, participants answered two additional questions to check their understanding and remembering of the text content. Text comprehension was checked with a question about the text content. Each reader had three answers possible, from which they chose the one they thought was correct. Text memorisation was checked by presenting the readers with a sentence and asking them whether they had read that exact sentence in the text. They also rated the pleasantness of the typeface with which the text was displayed, using a 5-point rating scale (1 – very unpleasant, 2 – unpleasant, 3 – neutral, 4 – pleasant, 5 – very pleasant).

#### 2.3.4. Data analysis

We considered the rating of pleasantness as a subjective measure of emotional response to typefaces, and pupil size as an objective measure of such response. Pupil size should be enlarged when a person experiences or perceives something pleasant (Hess and Polt, 1960; Margareth et al., 2008; Wang et al., 2018). Although pupil size under controlled lighting conditions may reflect factors other than the reader’s emotional response, such as surprise (Preuschoff et al., 2011) or cognitive load and metacognitive confidence (Gavas et al., 2018), we assume that these effects were minimized due to careful selection of texts in the preliminary study. We examined left pupil size (pupil diameter measured in millimeters). Pupil size changed during reading, but a careful examination of how it changed over time did not reveal specific patterns that could be generalized across different texts within a single participant or across different participants reading the same text. The 5-percent trimmed means of pupil diameter during the total time of reading a given text, which would eliminate potential outliers, were not significantly different from the uncorrected mean values (the difference was to the third decimal place), so we decided to use an uncorrected mean value of pupil diameter during the reading interval in further analyses.

Two objective indicators of reading fluency were analyzed, namely the number of regressive saccades and reading speed. Reading speed was determined by measuring the time spent per character (excluding spaces). Text comprehension and short-term text memory were used as measures of reading performance.

Data were analyzed using linear mixed modelling in the GAMLj module (Gallucci, 2020) for jamovi (The Jamovi Project, 2019).

To determine the extent to which pupil size actually reflects emotional response (typeface pleasantness), we first examined the relationship between subjective and objective indicators of emotional response to reading. Pupil size was used as an interval outcome variable, and pleasantness ratings centred within subjects were used as an interval predictor in the linear mixed model. The data were nested within participants. Participants were entered in the model as random intercepts and slopes.

Next, six different linear mixed models were developed. In each model, one of the six measures (pleasantness ratings, pupil size, number of regressive saccades, reading speed, text comprehension score, and text memorisation score) served as the outcome variable. Eight texts (level-1 units) were nested within 35 participants (level-2 units). Typeface shape was used as a level-1 predictor, i.e., as a within-subject factor-type variable with two levels describing the shape of the typeface (0 – round/rounded vs. 1 – angular/pointed typeface shape). Age was used as a level-2 predictor, i.e., a between-subject factor-type variable describing the participant (0 – child vs. 1 – university student). Three fixed effects were entered in the prediction model: the effect of typeface shape, the effect of age, and the interaction between age and typeface shape. To account for the inter-individual differences in the measured outcome variables, participants were entered in the model as random intercepts. Because we expected the effect of typeface shape to differ across participants, we also included the random slopes for typeface shape in the model. Equation 1 shows the model for predicting the outcome variable (Y′).


(1) $$\begin{array}{c} Y^{’}=b_{0}+b_{1}•\mathrm{Age}+b_{2}•\mathrm{Typeface shape}\\ \;+b_{3}•\mathrm{Age}\times \mathrm{Typeface shape}+\left(\mathrm{Intercept|Participant}\right)\\ \;+\left(\mathrm{Typeface Shape|Participant}\right) \end{array}$$

To examine the effect of a factor (typeface shape or age) manipulation on each of the six outcome variables, we compared Bayes factors (BF) for different models. We used the default settings of the BayesFactor package (Morey and Rouder, 2022) to calculate the BFs. The package specifies the Jeffrey prior for the grand mean and error variance, uses the default setting for the multivariate Cauchy prior distributions (scale set to 0.5 and 1 for fixed effects and random effects, respectively), and does not explicitly model the correlation between random slopes and intercepts (van Doorn et al., 2021). There is a “lack of clarity and consensus about how to best conduct Bayesian model comparison when considering mixed effects” (van Doorn et al., 2021, p. 2). Because we assume that some inter-individual variability is intrinsically present in the level of outcome variables and in the effect of typeface shape, we decided to use the model without fixed effects but with random intercepts and slopes specific to subjects as *a reference model*. To test for a specific fixed effect, we compared the reference model with a model that included the fixed effect under study along with random intercepts and slopes for the participants. We first calculated Bayes factors for both the reference model (BF_r_) and the fixed-effect model under test (BF_t_). Both BFs compared the model to the Intercept (b0)-only model (model without random or fixed effects). We then calculated the BF_t_/BF_r_ ratio. The ratio obtained (BF) greater than 1 indicated that the fixed-effects model was preferred, and BF less than 1 indicated that the reference model, i.e., the random-effects-only model, was preferred and that no notable fixed effect was present.

## 3. Results

The aim of our study was to examine the effect of typeface shape and age on reading. Table 1 shows the regression parameters for the fixed effects in the models tested. Large interindividual differences (large ICCs, i.e., intraclass correlation coefficients) were found in the eye-tracking measures—pupil size, number of regressive saccades, and reading time per character. ICCs were much lower for pleasantness ratings, text comprehension score and text memorisation score. For these variables, intrapersonal variability (differences between the eight typefaces) was much larger than interpersonal variability (differences between participants). However, on the legibility measures, intraindividual differences were much smaller than interindividual differences, suggesting that the reading skills of our participants were relatively diverse. Some were less fluent readers in general, i.e., across all eight texts, whereas the others were more fluent readers of all texts.

**Table 1:** Effect of typeface shape and age group on different reading parameters (pleasantness rating, pupil size, number of regressive saccades, reading speed, text comprehension and memorisation).

Corr. figure	Source of variability	*b*	*SE*_b_	95% CI for *b* lower bound	95% CI for *b* upper bound	*t*	*df*	BF
3A	**Pleasantness rating**							
	ICC = 0.028, LRT(2) = 0.397, *p* = 0.820, BF for the full model = 889.50							
	Intercept	3.41	0.07	3.27	3.54	47.65	36.6	
	Typeface shape	−0.78	0.13	−1.05	−0.53	−5.96	118.3	5100.71
	Age	0.29	0.14	0.01	0.57	2.01	36.6	0.57
	Typeface shape × Age	0.23	0.26	−0.28	0.75	0.89	118.3	0.48
3B	**Pupil size (mm)**							
	ICC = 0.874, LRT(2) = 0.048, *p* = 0.976, BF for the full model = 0.08							
	Intercept	2.76	0.03	2.69	2.83	79.28	33.0	
	Typeface shape	−0.02	0.01	−0.04	−0.00	−2.11	238.5	0.66
	Age	−0.02	0.07	−0.15	0.12	−0.26	33.0	0.74
	Typeface shape × Age	−0.003	0.02	−0.04	0.03	−0.16	238.5	0.22
4A	**Reading time per character (ms)**							
	ICC = 0.802, LRT(2) = 13.3, *p* < 0.001, BF for the full model = 540.71)							
	Intercept	71.21	3.22	64.90	77.51	22.13	33.0	
	Typeface shape	4.85	1.72	1.48	8.22	2.82	33.0	5.20
	Age	30.98	6.43	18.37	43.59	4.82	33.0	408.44
	Typeface shape × Age	0.71	3.44	−6.03	7.45	0.21	33.0	0.29
4B	**Number of regressive saccades**							
	ICC = 0.587, LRT(2) = 0.412, *p* = 0.814, BF for the full model = 0.02							
	Intercept	86.48	7.94	70.93	102.0	10.90	33.0	
	Typeface shape	1.74	4.54	−7.16	10.6	0.38	207.6	0.18
	Age	8.47	15.87	−22.64	39.6	0.53	33.0	0.43
	Typeface shape × Age	−2.55	9.08	−20.35	15.3	−0.28	207.6	0.22
5A	**Text comprehension**							
	ICC = 0.135, LRT(2) = 10.00, *p* = 0.007, BF for the full model = 0.13							
	Intercept	0.87	0.03	0.82	0.92	32.66	35.2	
	Typeface shape	−0.09	0.04	−0.17	−0.00	−2.02	53.2	0.85
	Age	−0.09	0.05	−0.20	0.01	−1.76	35.2	0.67
	Typeface shape × Age	0.00	0.08	−0.16	0.17	0.05	53.2	0.23
5B	**Text memorisation**							
	ICC = 0.072, LRT(2) = 0.53, *p* = 0.764, BF for the full model = 0.02							
	Intercept	2.62	0.04	2.53	2.70	62.28	33.3	
	Typeface shape	−0.09	0.07	−0.22	0.04	−1.33	123.6	0.28
	Age	−0.08	0.08	−0.25	0.08	−0.97	33.3	0.28
	Typeface shape × Age	−0.05	0.13	−0.32	0.21	−0.40	123.6	0.23

Corr. figure = corresponding figure number. LRT shows whether including the random slope in the model (i.e., random effect of typeface shape on the outcome variable, in other words the variability of the typeface shape effect across participants) improves the fit of the model, with all other model parameters held constant. BF shows the Bayes factors for the tested models with fixed effects and random effects (random slopes and intercepts) against the reference models with random effects only. BF larger than 1 indicates that the model with fixed effects was preferred, that is that the examined fixed effect was present, and BF smaller than 1 indicates that the model with random effects only was preferred, i.e., that no notable fixed effect was present.

No interaction between age and typeface shape was observed on any of the measures examined, so we can next focus on the main effects of typeface shape and age on various reading measures.

### 3.1. Effect of typeface shape and reader age on pleasantness ratings and pupil size

**Figure 2:**
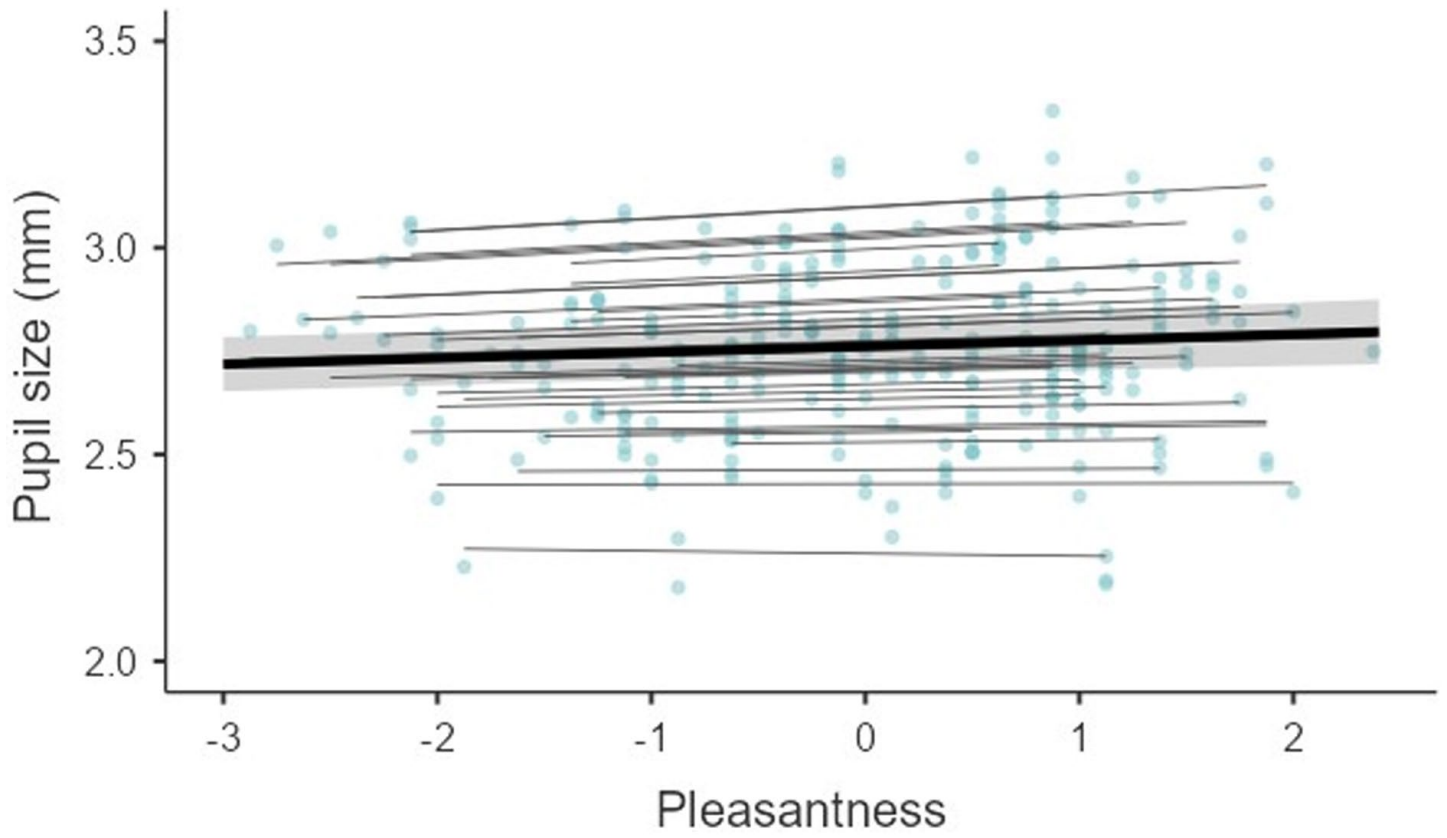
The relationship between pupil size and pleasantness ratings in different participants.

First, we examined the relationship between the subjective and objective indicators of the emotional response to reading. We found that ratings of pleasantness predicted pupil size (*b* = 0.01, β = 0.17, *SE*_b_ = 0.004, *t*(111.6) = 3.32), with strong support for the alternative hypothesis that the two variables are correlated (BF = 51.75). This suggests that readers responded emotionally to less or more pleasant typefaces. Pupil size was larger when reading typefaces were rated as more pleasant than when reading typefaces were rated as less pleasant (see also Figure 2).

Next, we examined the effects of typeface shape, age, and their interaction on pleasantness ratings and pupil size. Table 1 shows the results of linear mixed modelling and the Bayesian factors for each effect. BF values greater than 1 indicate evidence for the tested model, i.e., the model with both fixed and random effects, and values less than 1 indicate evidence for the reference model without fixed effects. In Table 1, we see that our data show very strong evidence for the effect of typeface shape on the pleasantness ratings. The extremely high BF value for the effect of typeface shape indicates that the model with fixed and random effects was preferred to the model with only random effects.

**Figure 3:**
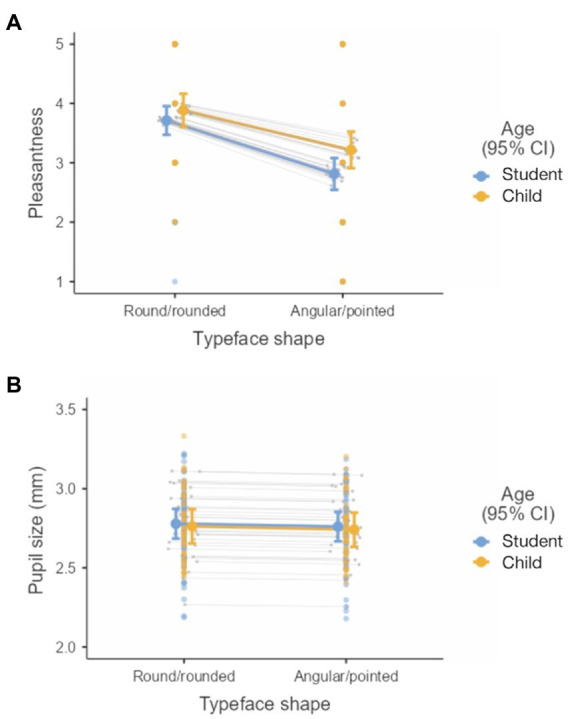
Effect of typeface shape and age group on measures of emotional response during reading: **(A)** Typeface pleasantness ratings and **(B)** pupil size. 95% confidence interval for means in different experimental conditions are shown. The same note also applies to Figures 4, 5.

Figure 3A shows the pleasantness ratings and Figure 3B shows the pupil size under different experimental conditions. Typeface shape, as already mentioned, affected the ratings of pleasantness. In general, readers rated round/rounded typefaces as more pleasant than the angular/pointed ones. No such effect of typeface shape was observed in the pupil size data. Pupil size was only slightly larger for round/rounded typefaces than for angular/pointed typefaces (Figure 3B). Pleasantness ratings and pupil sizes were relatively similar in children and adults.

### 3.2. Effect of typeface shape and age group on reading speed and number of regressive saccades

**Figure 4:**
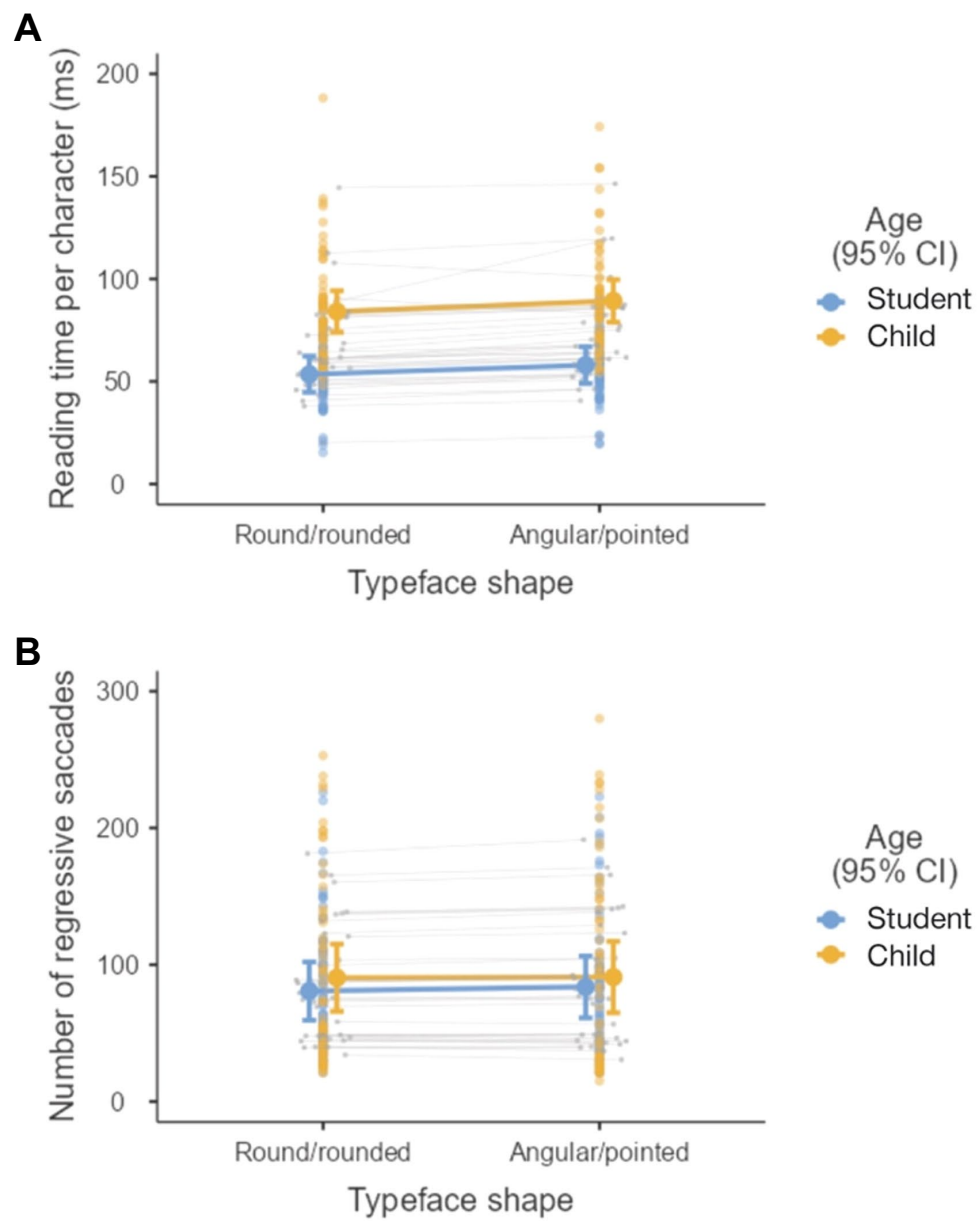
Effect of typeface shape and age group on measures of reading fluency: **(A)** Reading time per character and **(B)** number of regressive saccades.

Our data showed no evidence of a fixed effect of typeface shape on the number of regressive saccades; however, there was moderate evidence that typeface shape affected reading time per character (see Table 1; Figure 4A). The round/rounded typefaces had lower reading time per character than the angular/pointed typefaces. Thus, we can confirm that round/rounded typefaces allow for more fluent reading than angular/pointed typefaces.

An interesting discovery was that there was no fixed effect of age on the number of regressive saccades (see Table 1; Figure 4B). However, there was strong evidence for the effect of age on reading time per character (see Table 1; Figure 4A). Children read more slowly than adults.

### 3.3. Effect of typeface shape and reader age on text comprehension and text memorisation

**Figure 5:**
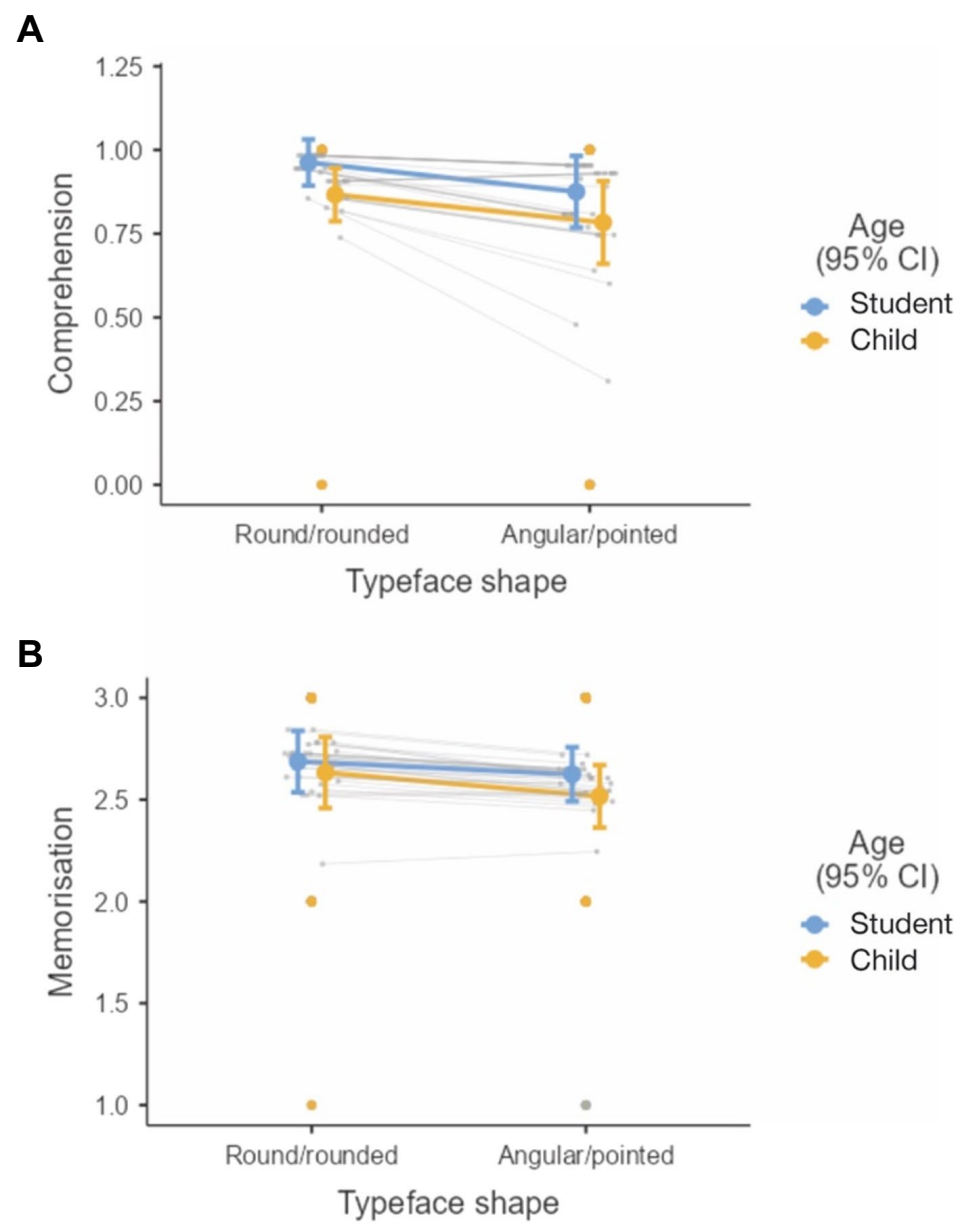
Effect of typeface shape and age group on reading performance measures: **(A)** Comprehension and **(B)** memorisation score.

Figures 5A,B show comprehension and memorisation scores under different experimental conditions, respectively. The analysis revealed no fixed effects of typeface shape, age, or their interaction on text comprehension or memorisation beyond the random effects. The BF values were in favour of the models with only random effects.

## 4. Discussion with conclusion

The aim of our study was to (i) determine how the shape of the typeface (round/rounded vs. angular/pointed) affects the feelings of pleasantness of the typeface and pupil size, reading fluency (reading speed and number of regressive saccades), and reading performance (text comprehension and memorisation), and to (ii) examine whether the effect of the shape of the typeface is the same for younger (less experienced) and older (more experienced) readers.

With regard to the second aim of our study, the absence of the interaction between age and typeface shape in all models tested showed that the effect of the shape of the typeface was the same for both age groups. With regard to the first aim of our study, we can conclude that the only notable fixed effects were the main effect of typeface shape on pleasantness ratings and reading speed and the main effect of age on reading speed. Other measures were better explained by the regression model which included only random intercepts and slopes. There was a great deal of variability in the measures examined between participants, either in their average level of the measures or in the effect of typeface shape on the measures.

### 4.1. Effect of typeface shape on examined parameters of reading

The pleasantness of the typeface was tested with a hedonic scale in which readers rated how pleasant they found the typeface. Both children and adults found round/rounded typefaces more pleasing than angular/pointed typefaces (see Table 1; Figure 3A).

The effect of different typeface shape on subjective experience was also tested by measuring pupil size while reading different typefaces. The measured pupil size was slightly larger when reading round/rounded typefaces (this can also be seen in Figure 3B), which was also perceived as more pleasant by the readers. The rated typeface pleasantness correlated with pupil size (see Figure 2), supporting the assumption that the shape of the typeface influences the reader’s emotional experience (Hess and Polt, 1960; Margareth et al., 2008; Wang et al., 2018). However, only the fixed effect of typeface shape on pleasantness ratings was convincing, whereas the effect of the typeface shape on pupil size was less remarkable. The analysis indicated that small differences in pupil size when reading round/rounded and angular/pointed typefaces could be a consequence of interindividual differences and could be attributed to random effects, i.e., to individual differences in pupil size and interindividual variability in the effect of typeface shape on pupil size. The fact that the effect of typeface shape on pupil size was smaller than effect of typeface shape on pleasantness ratings might indicate that factors other than the reader’s emotional response, e.g., surprise (Preuschoff et al., 2011) or cognitive load (Gavas et al., 2018), influenced pupil size, although we tried to control for cognitive load by selecting texts with homogeneous difficulty.

We found that the shape of the typeface had an effect on one of the measures of reading fluency, i.e., reading speed. Readers read round/rounded typefaces faster than angular/pointed typefaces (see Table 1; Figure 4B). Typeface shape did not show notable effects on other measures of reading fluency and reading performance measures. It is possible that our comprehension and memory tests were not discriminative enough to detect differences between the two typeface shapes. Future studies should use psychometrically validated measures of memorisation (and comprehension) for the texts used in the study.

Based on the results of our study, we can conclude that the shape of the typeface can influence reading speed and feelings of pleasantness while reading. Round/rounded typeface shapes may be perceived as more pleasant than angular/pointed shapes. Round/rounded typefaces also support reading fluency and allow readers to read faster.

### 4.2. Differences between age groups

Reading time varied by reader age – as expected, children read more slowly than adults, who tend to be more experienced readers (see Table 1; Figure 4B).

A somewhat surprising result was that the number of regressive saccades during the reading was not affected by age; that is, children did not have, on average, a higher number of regressive saccades than adults, as would be expected given their reading experience (see Table 1; Figure 4A). There were also no major differences between children and adults in text comprehension and memorisation. This can probably be explained by the fact that the texts used were not complex; they were easy to read and could be processed easily by both age groups. Future studies should examine how different reading parameters change with increasing text difficulty and whether age interacts with text difficulty in predicting reading performance and emotional and physiological responses during reading.

### 4.3. Limitations

Our study had several limitations. Even though we used texts of comparable difficulty, factors other than typeface shape may have influenced the results.

First, different participants might have responded differently to different texts. Their emotional response might depend on their specific interests (e.g., adults might respond differently to descriptions of animals than children). This could have increased the between-subject variability of the data.

Second, the typefaces we used differed in some characteristics that could affect reading parameters, such as typographic tonal density and overall character size: for example, we controlled for the x-height, but the different typefaces had different sizes of ascenders and descenders. As a result, the whiteness in the ascenders and descenders of the different typefaces was different, resulting in different line spacing, even though the leading was set to the same size (e.g., to 140%). Because of the different whiteness in ascenders and descenders, and because of the different counter shapes of the letters of different typefaces, the typographic tonal density value of texts in different typefaces will always be different, even if we unified the size of the x-height. Previous studies (Franken et al., 2015; Pušnik et al., 2016) have shown that factors such as these can affect reading speed and letter recognition. Future studies should investigate how manipulating a single feature of the typeface (e.g., only the shape of the strokes, while controlling for all other features, if possible) affects reading.

Third, the COVID-19 pandemic made it difficult to include larger samples, and the power of our complex statistical tests was low. Future studies should include larger samples.

Nevertheless, we believe that our results, although they should be considered preliminary, are quite informative because different measures of text processing were used, and although the fixed effects studied did not appear to be salient, all results pointed in the same direction – reading was more pleasant and fluent, and reading performance was minimally better with round/rounded typefaces compared to angular/pointed ones. Further studies will need to be conducted to provide more evidence, but our results suggest that it is important to consider typeface shape when examining reading or comparing findings from different studies.

### 4.4. Conclusion

Based on the results of our study, the use of round/rounded typefaces is recommended for the design of educational materials because readers or learners experience more pleasant feelings when reading than with angular/pointed typefaces. Using round/rounded typefaces also allows learners to read faster, which can have a positive impact on the learning process. The effect of typeface shape was similar in primary school pupils and university students, showing that the effect of typeface shape can be generalised across ages for simple texts. The typefaces with round/rounded shapes could be recommended for the design of educational materials used on the screen of a digital device for less experienced and more experienced readers. Such typefaces could make the learning process easier and more enjoyable.

## Data availability statement

The raw data supporting the conclusions of this article will be made available by the authors, without undue reservation.

## Ethics statement

The studies involving human participants were reviewed and approved by Ethics Commission of the Faculty of Arts, University of Ljubljana. Written informed consent to participate in this study was provided by the participants' legal guardian/next of kin.

## Author contributions

TM is a PhD student who has conducted research as part of her dissertation that she plans to publish in the indicated article. KM is her mentor and AP is her co-mentor. TM, KM, and AP jointly prepared a research plan for preliminary studies and for the final study. TM conducted all studies. TM reviewed and collected the literature. AP assisted TM in preparing data for statistical analysis. AP and TM jointly prepared the statistical analysis of the streams. All authors contributed to the article and approved the submitted version.

## Funding

The authors acknowledge the financial support from the Slovenian Research Agency (research core funding no. P5-0110 and no. P2-0213, and Infrastructural Centre RIC UL-NTF).

## Conflict of interest

The authors declare that the research was conducted in the absence of any commercial or financial relationships that could be construed as a potential conflict of interest.

## Publisher’s note

All claims expressed in this article are solely those of the authors and do not necessarily represent those of their affiliated organizations, or those of the publisher, the editors and the reviewers. Any product that may be evaluated in this article, or claim that may be made by its manufacturer, is not guaranteed or endorsed by the publisher.
