# Supplementary material for: Influence of letter shape on readers’ emotional experience, reading fluency, and text comprehension and memorisation
Source: Front Psychol. 2023 Feb 15;14:1107839. doi: 10.3389/fpsyg.2023.1107839 (PMC9996753; doi:10.3389/fpsyg.2023.1107839)

**Influence of letter shape on readers’ emotional experience, reading fluency, and text comprehension and memorisation**

**Tanja Medved^1^, Anja Podlesek^2^, Klementina Možina^1^**

^1^ University of Ljubljana, Faculty of Natural Sciences and Engineering

^2^ University of Ljubljana, Faculty of Arts

**SUPPLEMENTARY MATERIALS**

Eight figures showing 8 texts in Slovenian language used in the main study set in typefaces that were used in the main study (type size 12 pt) and their translation in English language set in Helvetica Neue (type size 12 pt)

**Figure 6**

Text no. 3, Typeface no. 1 (Chaparral Pro)


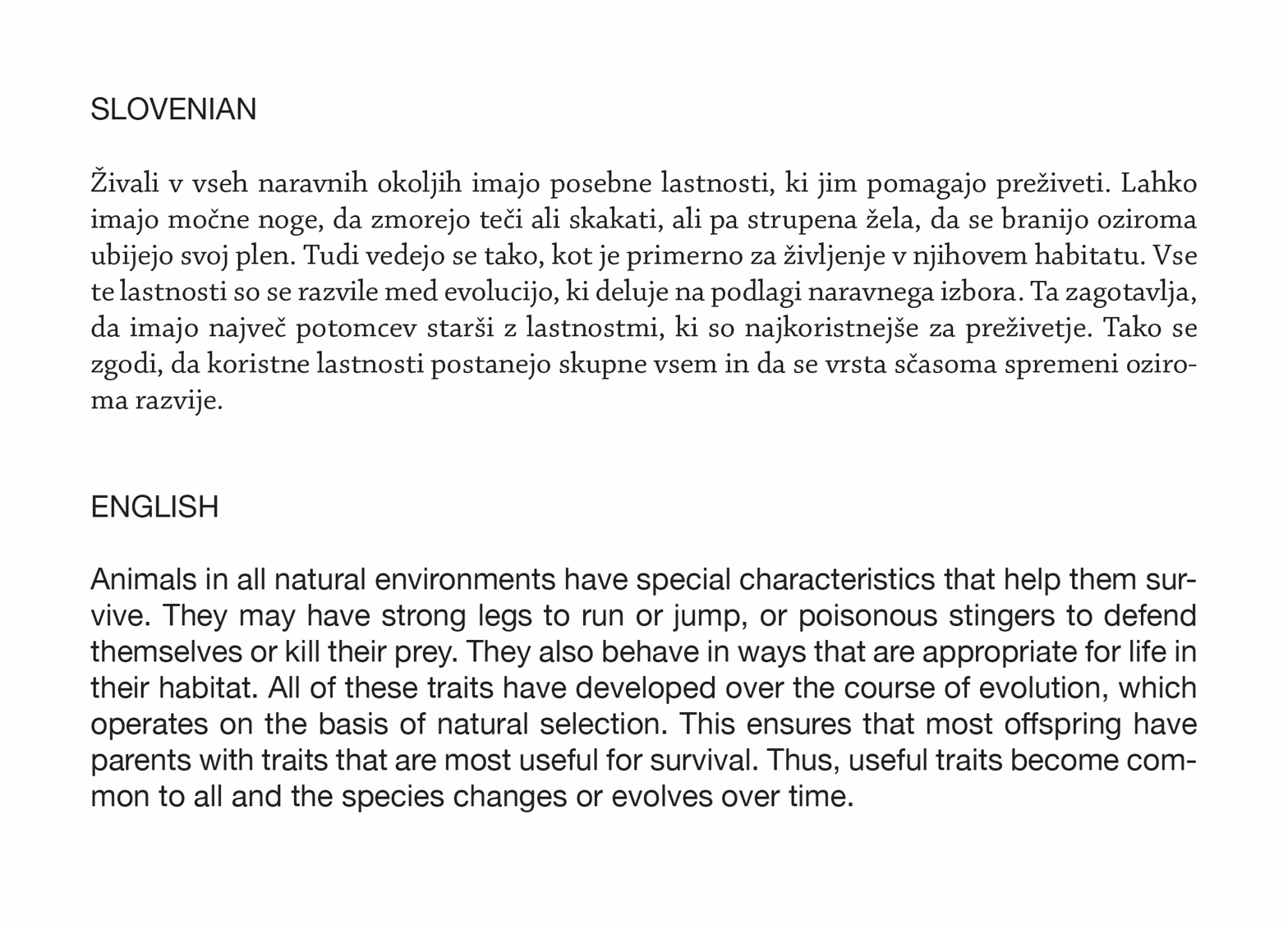


**Figure 7**

Text no. 10, Typeface no. 2 (Verdana)


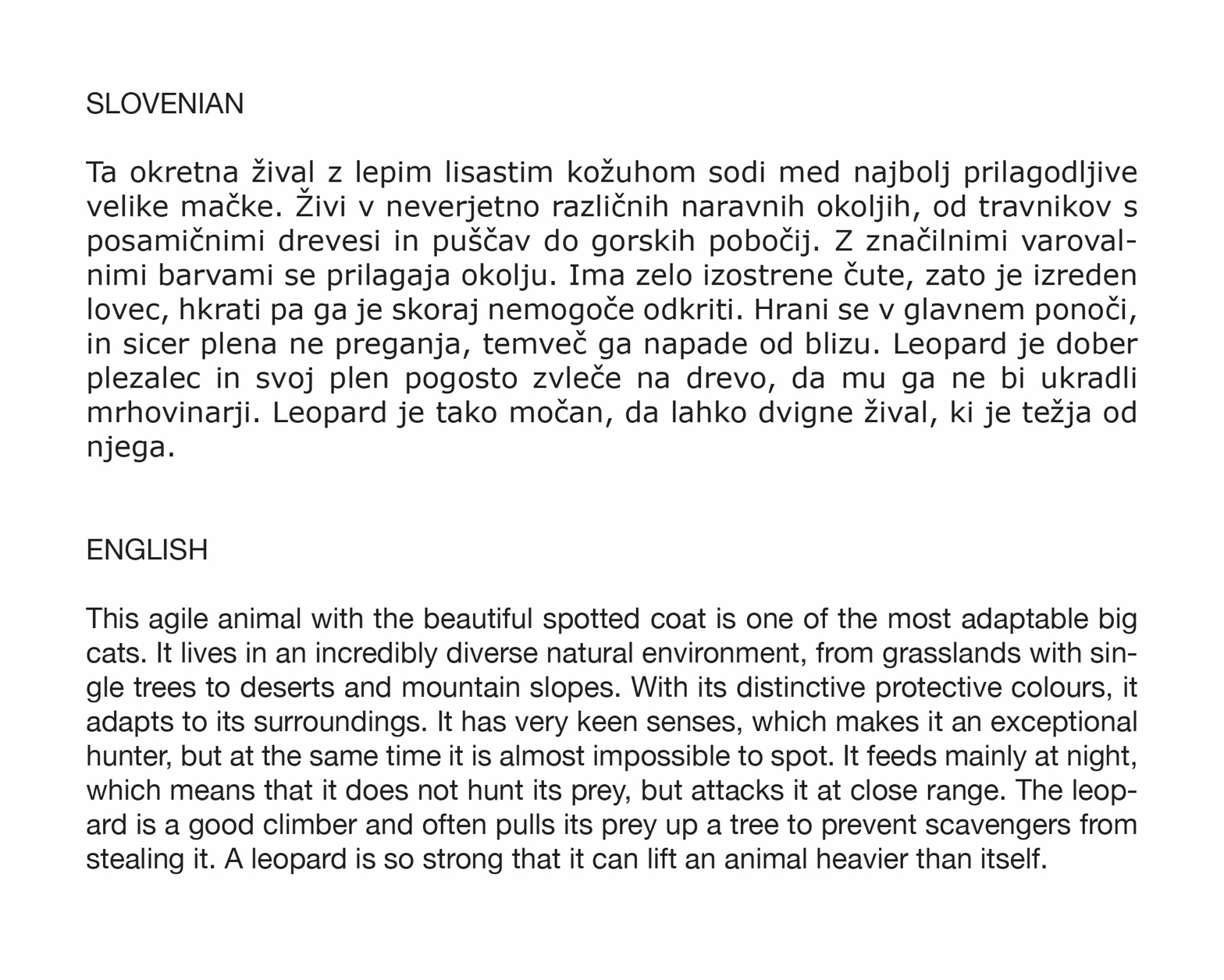


**Figure 8**

Text no. 17, Typeface no. 3 (Times New Roman)


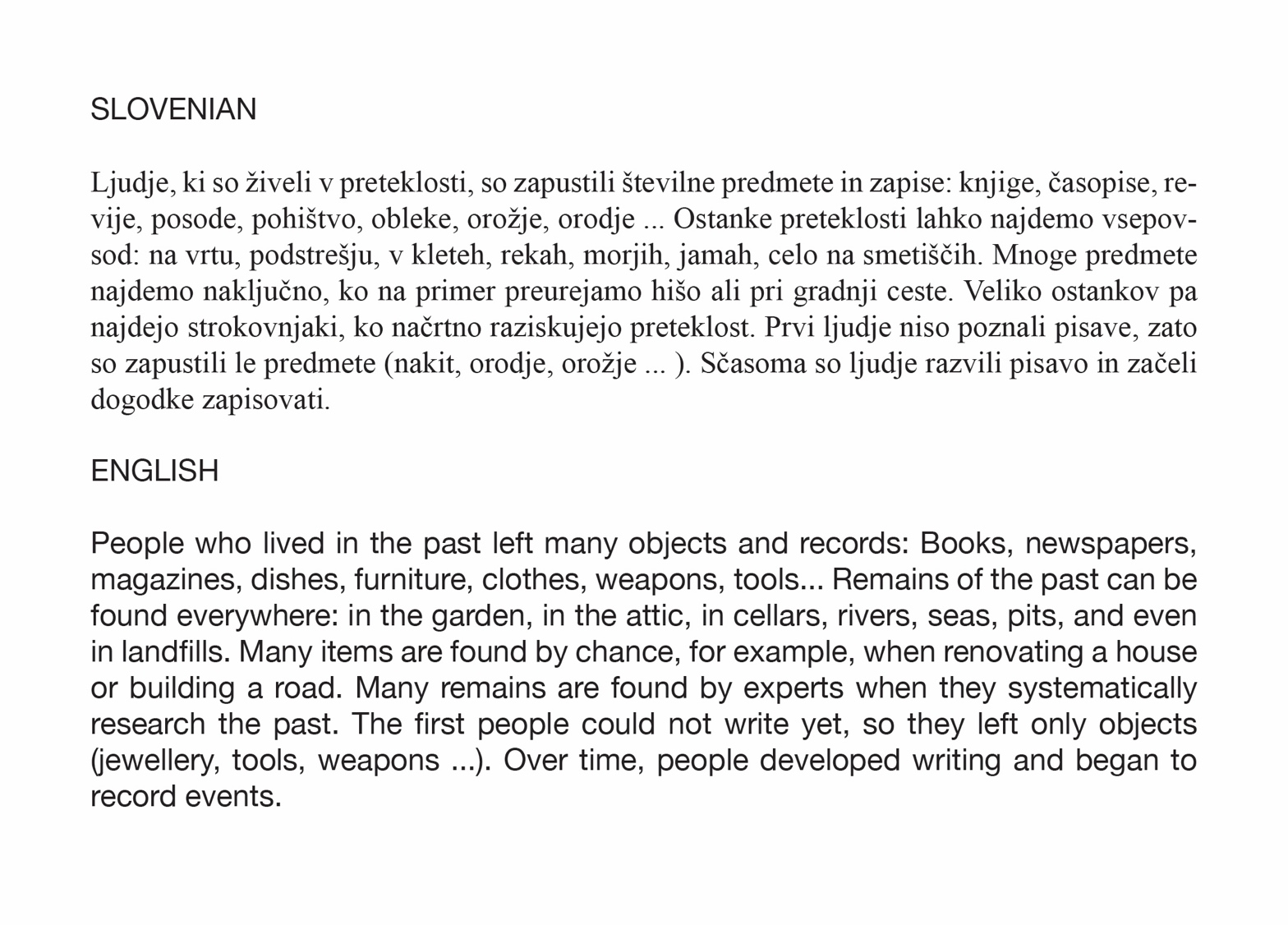


**Figure 9**

Text no. 21, Typeface no. 6: Arial Nova


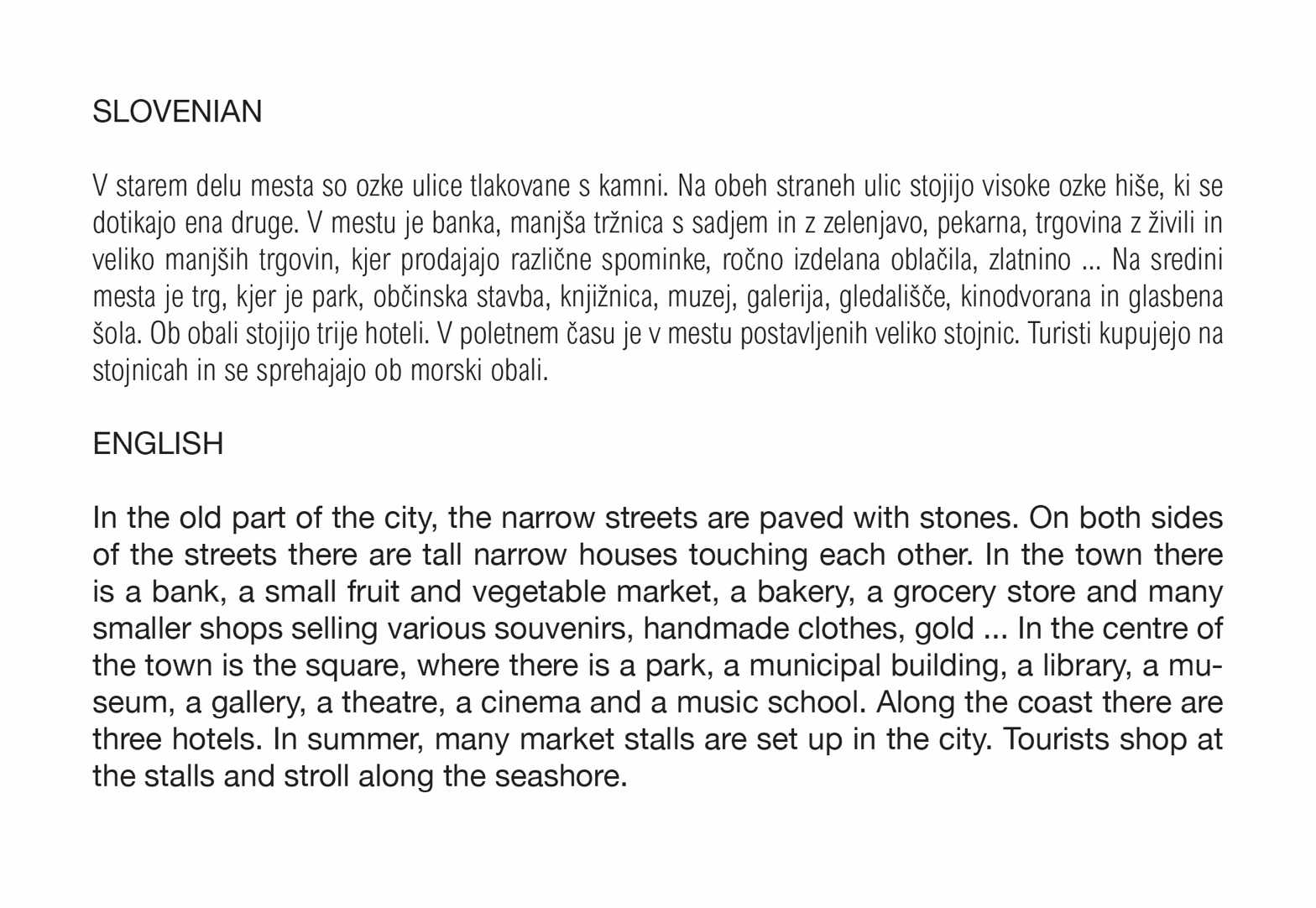


**Figure 10**

Text no. 22, Typeface no. 9 (Sans Forgetica)


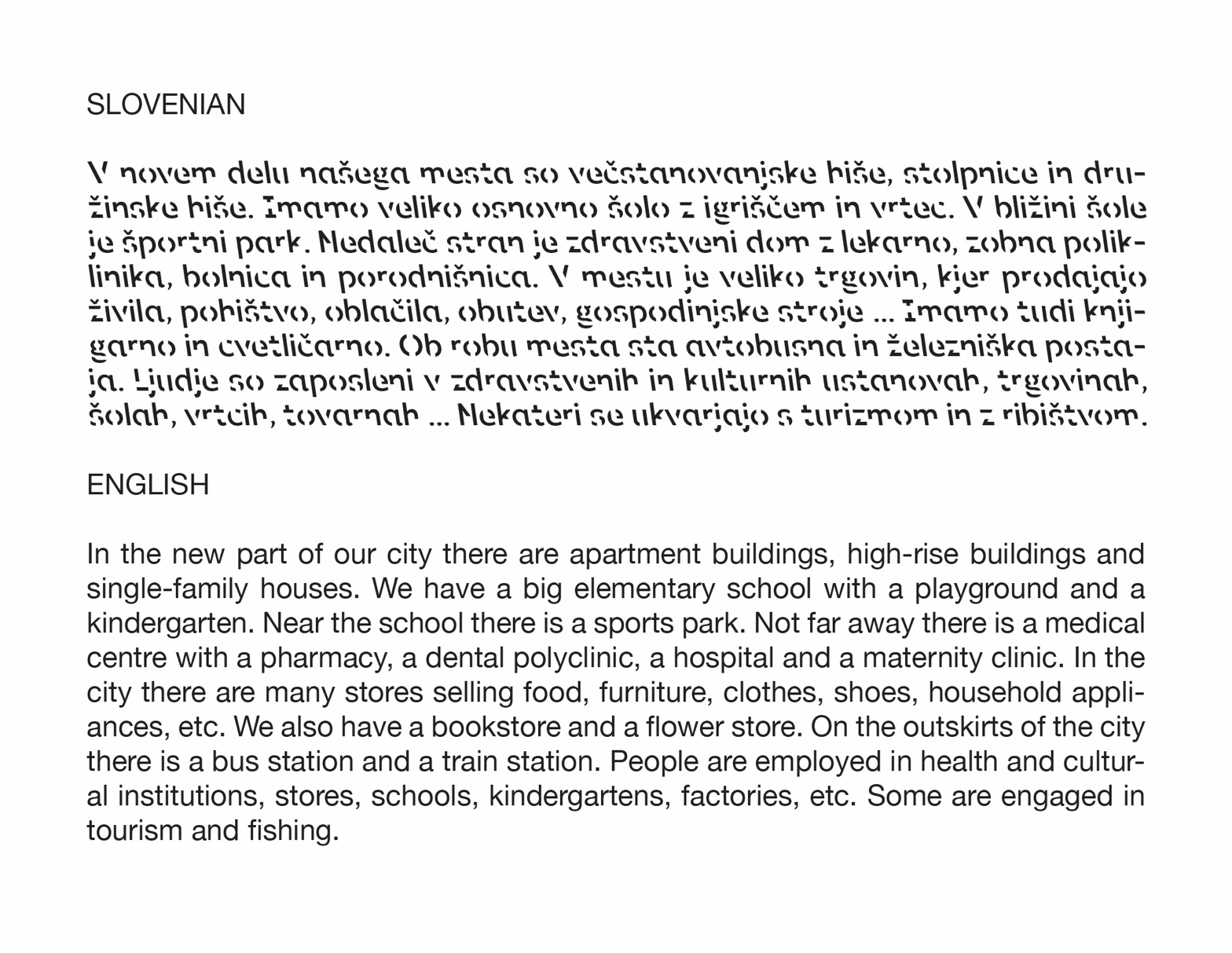


**Figure 11**

Text no. 23, Typeface no. 12 (Nogomet)


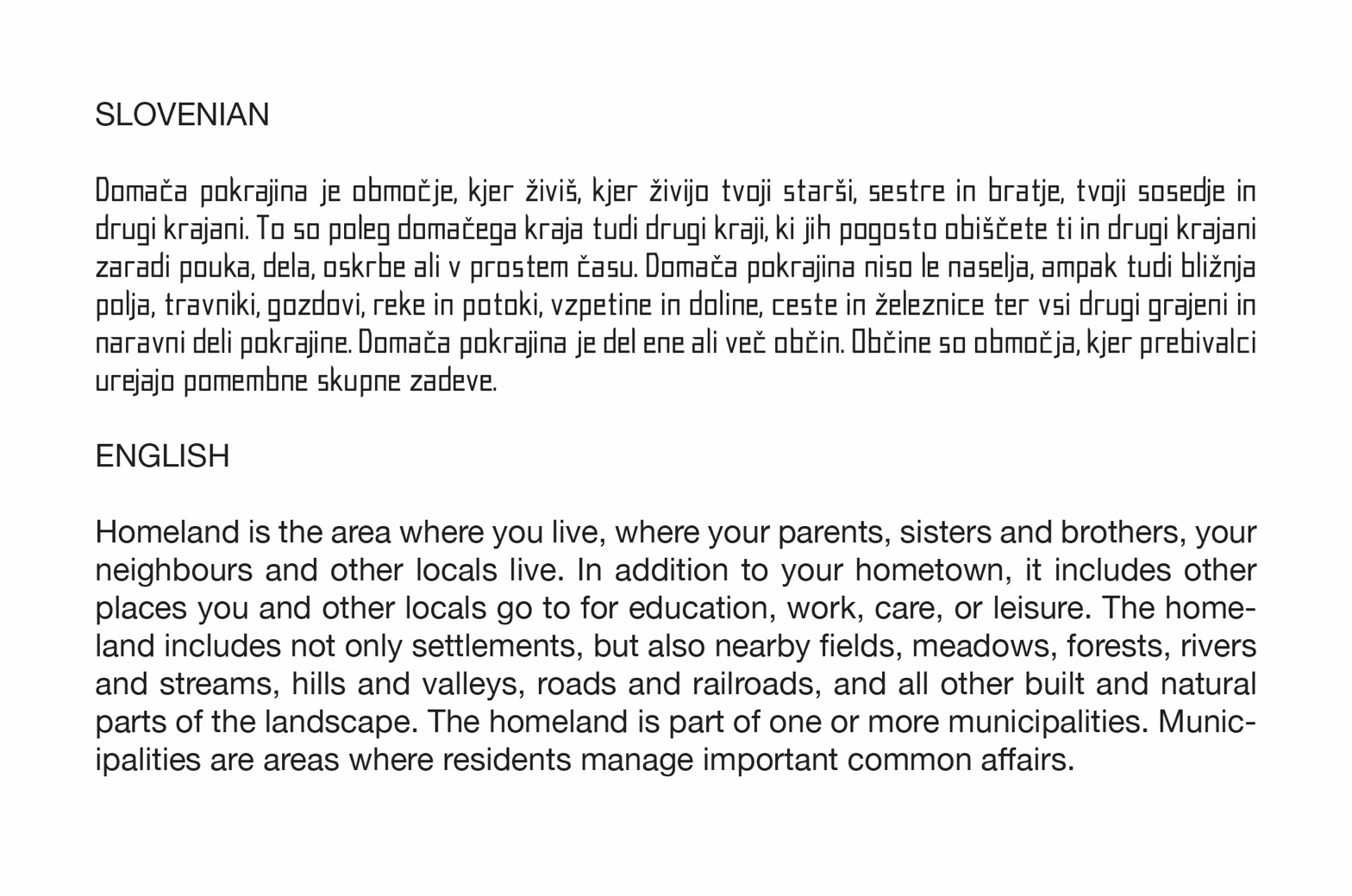


**Figure 12**

Text no. 35, Typeface no. 13 (FG April Trial)


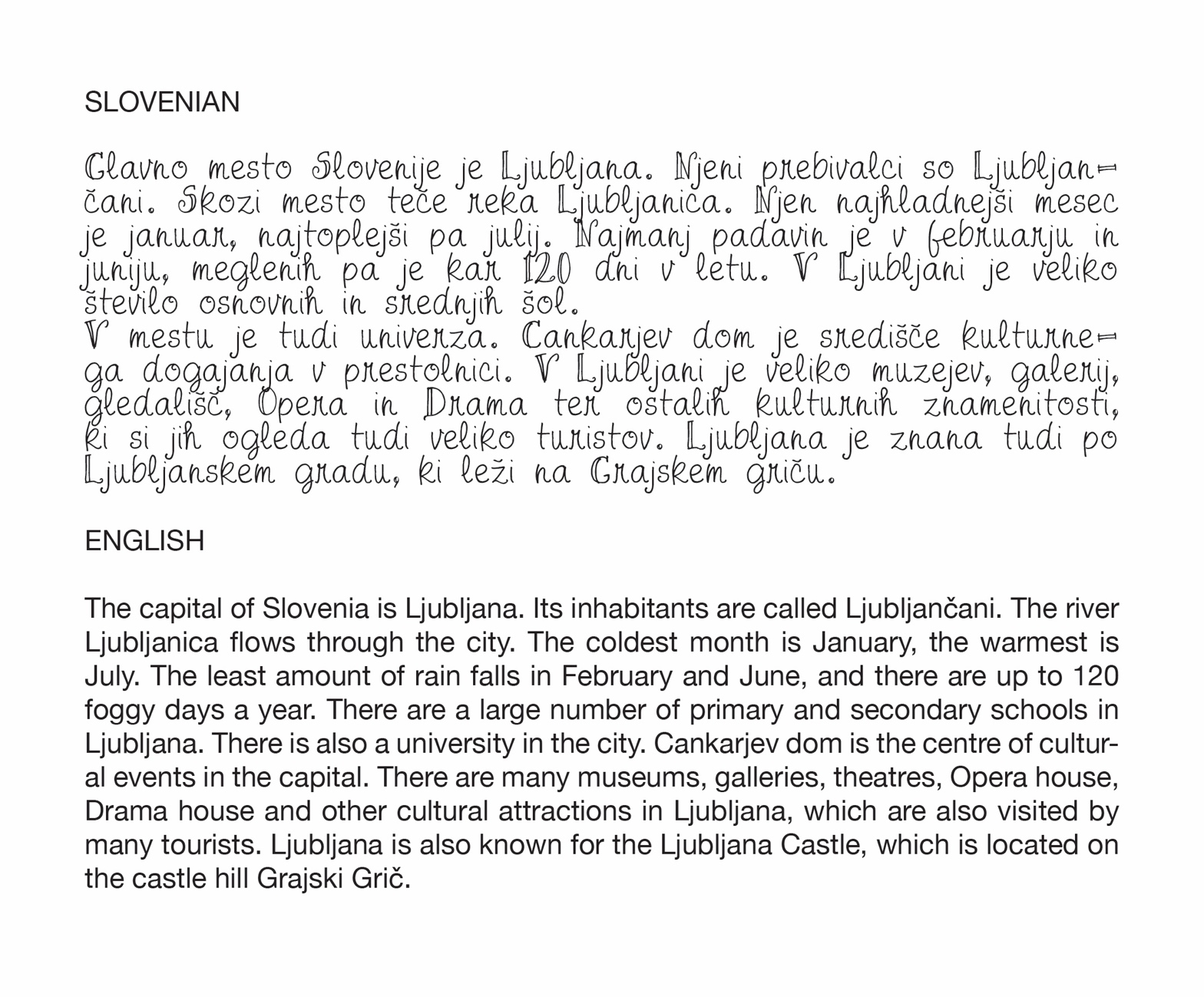


**Figure 13**

Text no. 40, Typeface no. 15 (Matilda)


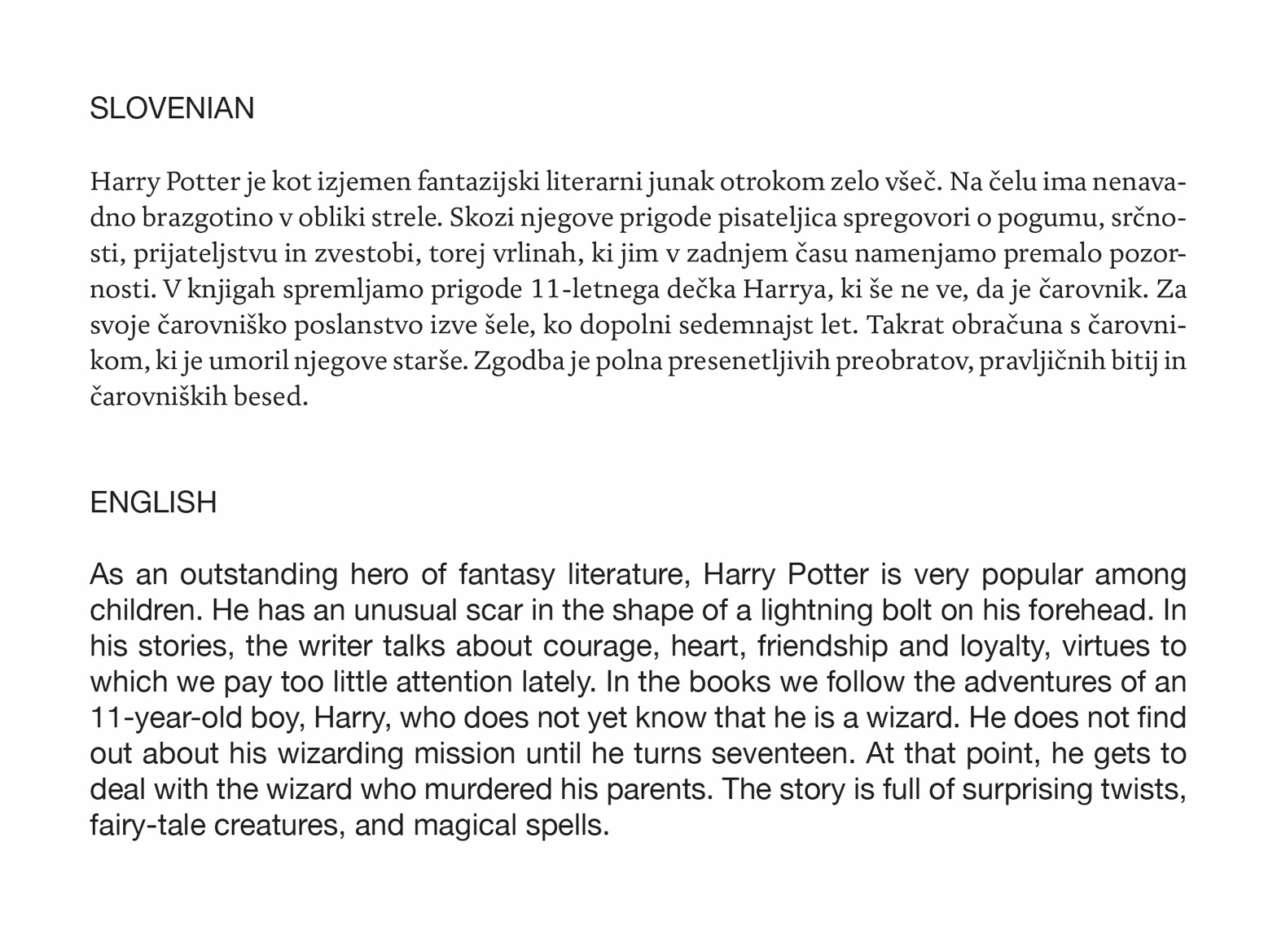

Supplement: Supplementary file 1 [file Data_Sheet_1.docx]
